# Severe early hepatitis B reactivation in a patient receiving anti-CD19 and anti-CD22 CAR T cells for the treatment of diffuse large B-cell lymphoma

**DOI:** 10.1186/s40425-019-0790-y

**Published:** 2019-11-21

**Authors:** Jia Wei, Xiaojian Zhu, Xia Mao, Liang Huang, Fankai Meng, Jianfeng Zhou

**Affiliations:** Department of Hematology, Tongji Hospital, Tongji Medical College, Huazhong University of Science and Technology, Wuhan, 430030 Hubei China

**Keywords:** Hepatitis B virus, Reactivation, Chimeric antigen receptor T-cell, Diffuse large B-cell lymphoma

## Abstract

**Background:**

Hepatitis B virus (HBV) reactivation is commonly seen in HBsAg-positive hematologic patients undergoing immunosuppressive chemotherapy. Little is known about the risk of HBV reactivation after chimeric antigen receptor T-cell (CAR T) immunotherapy for the treatment of refractory/relapsed malignant B-cell lymphoma.

**Case presentation:**

We report a patient who underwent antiviral prophylaxis for 26 months and who discontinued treatment by herself 1 month after the sequential infusion of two specific, third-generation anti-CD19 and anti-CD22 CAR T cell immunotherapies for refractory/relapsed diffuse large B-cell lymphoma. Remission of the primary disease was achieved after two and half months, but she was admitted with a 7-day history of vomiting, jaundice, itching and dark urine. After excluding other possible causes of acute liver damage, HBV reactivation was suspected. HBV-DNA was 4,497,000 IU/mL at that time. Following the reintroduction of entecavir, a decline in the HBV-DNA copies was observed, but ALT, AST and bilirubin were elevated, and there was no improvement of the clinical conditions. She passed away because of hepatic encephalopathy and multiple organ dysfunction syndrome 40 days after admission.

**Conclusions:**

Our study provides the first report of the severe, early reactivation of an inactive HBsAg carrier after CAR T cell therapy in DLBCL.

**Trial registration:**

ChiCTR-OPN-16008526.

## Background

Immunotherapy has become one of the most promising treatments for refractory/relapsed B cell lymphoma [1, 2]. Among immunotherapies, chimeric antigen receptor T (CAR T) cell immunotherapy has recently been found to be a highly effective treatment for common pre-B cell acute lymphoblastic leukemia and for relapsed or refractory diffuse large B-cell lymphoma (DLBCL), resulting in approximately a 40% durable response [3–6]. Our preliminary unpublished results showed that sequential infusion of CAR 19/22 T-cells is safe and well tolerated in patients with refractory/relapsed B-cell malignancies. The safety of CAR T cell therapy and the risk of the reactivation of hepatitis B virus (HBV) in DLBCL patients who are HBV inactive carriers (HBsAg-positive with undetectable HBV-DNA) has not yet been assessed.

The reactivation of HBV is a well-known complication in patients undergoing chemotherapy or immunosuppressive therapy for hematologic malignancies, particularly in the event of stem cell transplantation or when using monoclonal antibodies against the CD20 protein, which is found on the surface of immune system B cells, such as rituximab [7–10]. The reactivation of HBV is defined as a more than 10-fold increase in HBV-DNA, the detection of HBV-DNA in a patient who previously had undetectable HBV-DNA, or when reverse seroconversion occurs with liver damage, which is seldom life-threatening [11]. Guidelines suggest that antiviral prophylaxis should be initiated at least 1 week before or when starting chemotherapy. Antiviral prophylaxis should be continued for the duration of chemotherapy and should be administered for at least 12 to 24 months after the discontinuation of the immunosuppressive regimen [12]. No guidelines are available that provide a clear consensus regarding the management of patients with resolved HBV infections undergoing CAR T cell therapy. The safety of CAR T cell therapy in patients with B-cell lymphoma and HBV infection remains completely unexplored. Here, we report a case of early HBV reactivation in a patient diagnosed with diffuse large B-cell lymphoma who was treated with the sequential infusion of anti-CD 19 and anti-CD 22 CAR T cells.

## Case report

A 64-year-old woman was diagnosed with diffuse large B-cell lymphoma at the IIIB stage (Ann Arbor staging system) 5 years ago and received a standard dose of R-CHOP (rituximab, cyclophosphamide, vincristine, adriamycin and prednisone) for 8 cycles and achieved complete remission. She had an enlargement of the cervical lymph nodes and suspected remission four and half years after initial diagnosis. The patient underwent rebiopsy of the cervical lymph nodes. The pathology revealed a relapse of the primary disease. Next-generation sequencing (NGS) of the resected lymph nodes revealed a CARD11 K215 T mutation without any other mutations. After relapse, she received a standard dose of R-ICE (rituximab, ifosfamide, carboplatin, and etoposide) for 2 cycles and intermittently took lenalidomide, but the disease still progressed. She had a history of HBV infection, and blood tests were positive for HBsAg, anti-HBc and anti-HBe, with undetectable serum HBV-DNA levels. Anti-hepatitis C virus (HCV) antibody results were negative. Serum aspartate aminotransferase (AST) and alanine aminotransferase (ALT) were consistently normal, and no hematochemical and liver ultrasound findings were indicative of chronic active hepatitis. The patient received antiviral prophylaxis with entecavir (0.5 mg per day) during chemotherapy and had discontinued antiviral prophylaxis 1 year ago.

**Fig. 1:**
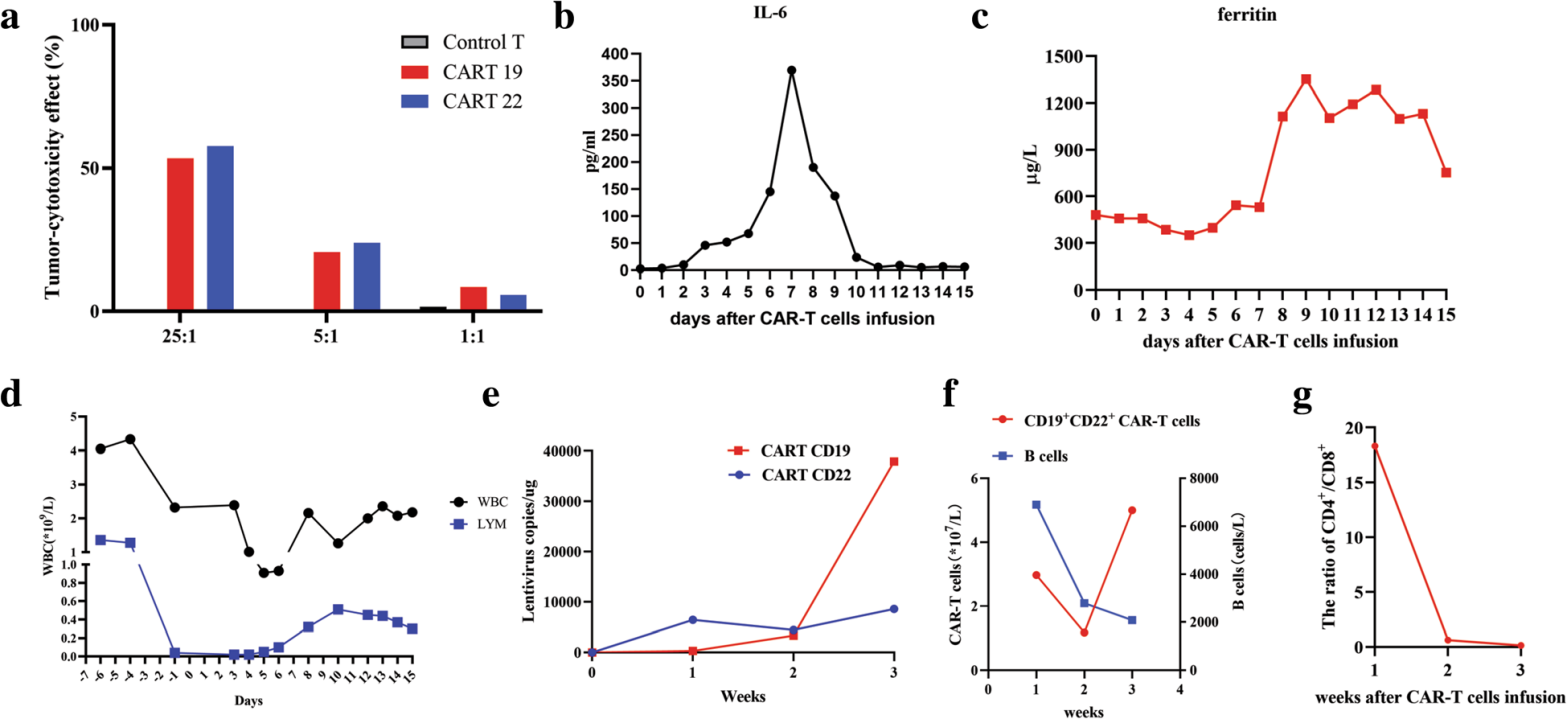
Sequential infusion of anti-CD 19 and anti-CD22 CAR T cell therapy. **a** In vitro tumor-cytotoxicity effect of CART 19 and CART 22 cells at effector/target ratios of 25:1, 5:1 and 1:1. **b** Levels of IL-6 after CAR T cell therapy. **c** Levels of ferritin after CAR T cell therapy. **d** Dynamic white blood cell numbers and lymphocyte numbers before and after CAR T cell therapy. **e** Copies of lentivirus-containing CARs in the peripheral blood after CAR T cell therapy. **f** CAR T cell and B cell numbers after CAR T cell therapy. **g** The ratio of CD4+/CD8+ T cells in the peripheral blood after CAR T cell therapy

In the presence of relapsed disease, we tried to use CAR T therapy with anti-CD19 and anti-CD22 CAR constructs to generate CAR T 19 and CAR T 22 cells, respectively. Although the HBV-DNA level remained undetectable, we reintroduced entecavir (0.5 mg per day) 2 months before CAR T cell therapy. Autologous peripheral blood mononuclear cells (PBMCs) were cultured with an anti-CD3 monoclonal antibody to induce T cell proliferation. The anti-CD22 CAR T and anti-CD19 CAR T cells were cultured for 14 days before infusion. Subsequently, she was conditioned with a standard lymphodepleting chemotherapy regimen consisting of fludarabine (25 mg/m^2^) and cyclophosphamide (20 mg/kg) on day − 4 ~ − 2. The sequential infusion of CAR T cells was performed as follows: 4 × 10^6^ cells/kg of CAR T 22, divided into two infusions on day 0 to day + 1 (7/3/2018 and 7/4/2018), followed by 4 × 10^6^ cells/kg CAR T 19, divided into two infusions on day + 2 and day + 3 (7/5/2018 and 7/6/2018). The autologous CAR T cells proliferated in vitro, and the tumor-cytotoxic effect of CAR T 19 and CAR T 22 was up to 53.36 and 57.71%, respectively, with an effector/target ratio of 25:1 (Fig. 1a). After CAR T infusions, she had 1~2 degree of cytokine release syndrome with elevated IL-6 and ferritin. The levels of IL-6 and ferritin gradually returned to the baseline levels 2 weeks after CAR T therapy (Fig. 1b–c). On day + 10 after CAR T cell infusion, the WBC increased to 1.26 × 10^9^/L with 0.33 × 10^9^/L neutrophil granulocytes, 0.51 × 10^9^/L lymphocytes and 0.27 × 10^9^/L monocytes. The dynamic changes in white blood cells and lymphocytes after CAR T cell therapy are depicted in Fig. 1d. Lentivirus copies detected by PCR and CD19^+^CD22^+^ CAR T cells detected by flow cytometry from the CAR T cell infusion significantly increased with a decline in B lymphocytes, indicating that CAR T cells reached their peak levels after 3 weeks (Fig. 1e–f). In addition, the ratio of CD4^+^/CD8^+^ T cells in the peripheral blood was significantly below normal two and 3 weeks after CAR T cell infusion (Fig. 1g).

**Fig. 2:**
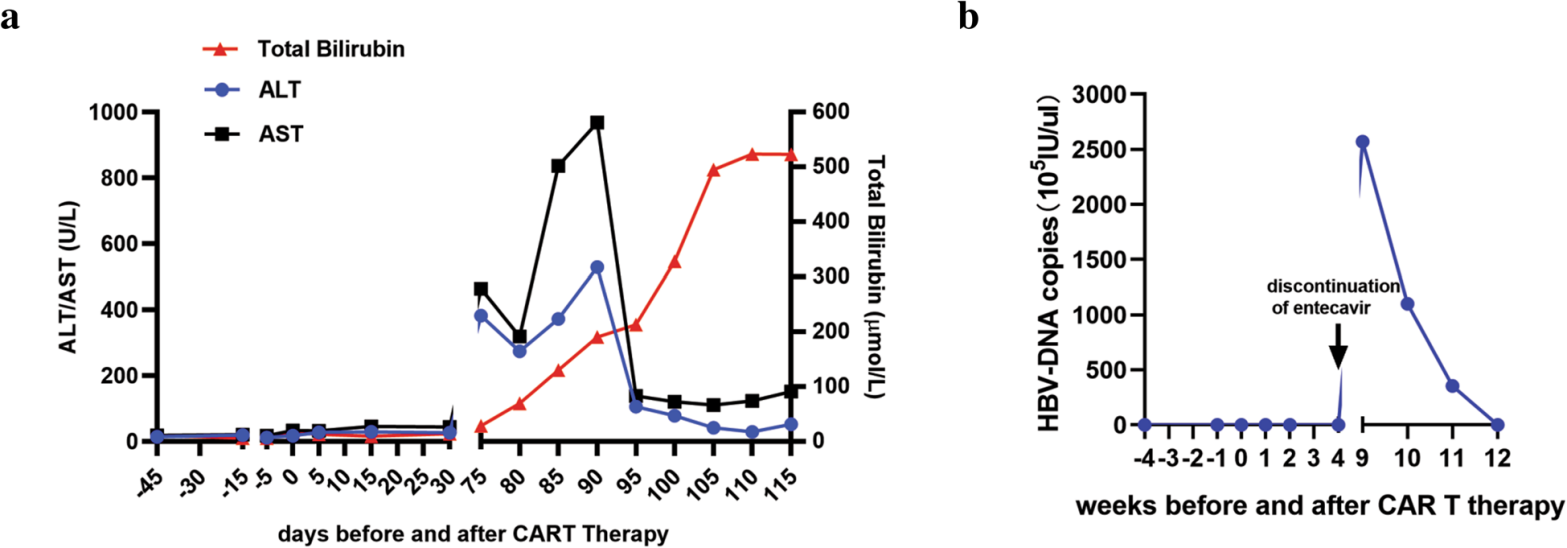
Longitudinal evaluation of hepatitis B virus (HBV)–DNA, liver enzymes, and bilirubin. **a** The dynamic changes in ALT, AST and total bilirubin before and after CAR T cell therapy. **b** HBV-DNA copies before and after CAR T cell therapy

The patient was instructed to remain on antivirals. The patient, however, became noncompliant and discontinued entecavir 1 month after CAR T immunotherapy. She was admitted with a 7-day history of vomiting, jaundice, itching, and dark urine two and half months after CAR T cell therapy. The primary disease was stable two and half months after treatment. No superficial lymph nodes could be palpated. Cervical lymph node and abdominal ultrasonography revealed that no superficial and deep lymph nodes could be detected. The evaluation of CAR T therapy by flow cytometry revealed 1.4% CD19^+^CD22^+^ CAR T cells. After excluding other possible causes of acute liver damage, HBV reactivation was suspected. Blood tests were positive for HBsAg, anti-HBc HBeAg and anti-HBe. The level of HBV-DNA was 2.57 × 10^8^ IU/mL. Anti-HAV IgM, anti-HCV and anti-HIV showed negative results. The blood tests performed after admission showed elevated ALT and AST. BUN and creatinine were normal at that time. No ascites was found by ultrasonography. Following the reintroduction of entecavir (1 mg once daily), a decline in HBV-DNA copies was observed, but the ALT, AST and bilirubin levels continued to increase (Fig. 2a–b), and there was no improvement in the clinical condition of the patient. The patient was then transferred to the department of infectious disease. Plasma exchange therapy with an artificial liver support system was used periodically every five to 6 days. The patient soon entered a stage of hepatic coma, and the phenomenon of enzyme bilirubin separation was observed, which is associated with poor outcomes. Unfortunately, her symptoms worsened. She passed away because of deteriorated hepatic function 40 days after admission. The brief chronology of the key clinical events in this case is depicted in Table 1.

**Table 1:** Brief chronology of the key clinical events in this case

Time before and after CAR T cell therapy	Date	Key events
−5 years	2014/7	Diagnosis of DLBCL
−5 to −0.5 years	2014/7 to 2018/1	8 cycles of R-CHOP and sustained remission
−1 year	2017/7	Discontinuation of entecavir
−6 months	2018/1	Relapse/reintroduction of entecavir
−4 days to −2 days	2018/6/29 to 2018/7/1	Standard lymphodepleting chemotherapy regimen
0 days	2018/7/3	Start of CART therapy
0 days to + 3 days	2018/7/3 to 2018/7/6	Sequential infusion of anti-CD 19 and anti-CD22 CAR T cell therapy
+ 4 weeks	2018/8/4	Discontinuation of entecavir
+ 9 weeks	2018/9/4	Activation of hepatitis B
+ 3.3 months	2018/10/12	Death

## Discussion

Adoptive cellular immunotherapy with chimeric antigen receptor (CAR) T cells has changed the treatment landscape of B-cell non-Hodgkin’s lymphoma (NHL), especially for aggressive B-cell lymphomas [5, 13, 14]. While CAR T cell therapy has a promising future in the treatment of lymphoma in general and particularly in the treatment of aggressive lymphoma, there is still a chance of failure. We observed severe, early HBV activation in a patient who received CAR T cell therapy.

HBV is a double-stranded DNA virus that induces a host immune response in hepatocytes via MHC II-CD4^+^ helper T cells and MHC I-CD8^+^ cytotoxic T cells. HBV reactivation is commonly observed in HBsAg-positive patients undergoing immunosuppressive anticancer therapy; targeted therapies with monoclonal antibodies and rituximab-containing chemotherapy for hematologic malignancies have been recognized as risk factors for HBV reactivation among both active and inactive HBsAg carriers. The highest rates of reactivation are typically observed during immunochemotherapy with the anti-CD 20 monoclonal antibody rituximab, especially when this treatment is combined with cyclophosphamide, doxorubicin, vincristine and prednisone chemotherapy [8, 9, 15, 16]. HBV DNA monitoring–guided preemptive nucleos(t) ide therapy can prevent HBV hepatitis during anti-CD20 immunochemotherapy in B-cell NHL [7]. Although there have been few reports of antiviral prophylaxis for HBV reactivation in patients with CAR T cell therapy [17], universal prophylaxis is recommended in selected clinical settings, such as long-term immunosuppression.

Current guidelines recommend that patients who are HBV inactive carriers (HBsAg-positive with undetectable HBV-DNA) must start pre-emptive prophylaxis with an antiviral agent at the beginning of immunosuppressive therapy [18, 19]. The duration should continue for at least 12 months after cessation of the immunosuppressive treatment [18, 19]. It is important to screen all patients receiving CAR T therapy for evidence of chronic hepatitis B infection by testing for HBsAg, anti-HBcAb and anti-HBs. As B cell aplasia and T cell immune reconstitution can be prolonged after the sequential infusion of anti-CD 19 and anti-CD 22 CAR T cell therapy, antiviral prophylaxis may need to be continued for longer than 12 months to prevent HBV reactivation. Patients with active HBV infections were excluded from this clinical trial. However, our clinical trial did not exclude HBV inactive carriers. In this clinical trial, HBV carriers were instructed to take prophylactic antiviral treatment before CAR T therapy. It was suggested in the clinical trial that the patients should not stop prophylactic antiviral treatment until at least 6 months after the full recovery of B cells. The use of third-generation antiviral drugs (entecavir or tenofovir) is strongly recommended in HBsAg-positive patients regardless of the HBV DNA levels since there is 20 to 30% HBV reactivation breakthrough in patients receiving lamivudine [20]. Since detectable HBV DNA at baseline was strongly associated with an increased risk of reactivation, the periodic monitoring of HBV-DNA is extremely important in CAR T cell therapy. Prophylactic anti-HBV treatment should be continued before and for at least 12 months after the discontinuation of B-lymphocyte-targeting drugs; additionally, more data should be collected to define the exact duration of HBV prophylaxis in CAR T cell therapy.

In summary, this is the first report of the early reactivation of an inactive HBsAg carrier after CAR T cell therapy. More data should be collected to assess the incidence of HBV reactivation after CAR T cell therapy. The exact time of prophylactic anti-HBV treatment after CAR T cell therapy should also be defined.

## Data Availability

All published data and material are available upon request from the corresponding author.

## Abbreviations

ALT: Alanine aminotransferase
AST: Aspartate aminotransferase
CAR T: Chimeric Antigen Receptor T-Cell
DLBCL: Diffuse large b-cell lymphoma
HBcAb: Antibodies to hepatitis B core
HBsAg: Hepatitis B surface antigen
HBV: Hepatitis B virus
NHL: Non-Hodgkin’s lymphoma

## References

[1] Gisselbrecht C, Van Den Neste E (2018). How I manage patients with relapsed/refractory diffuse large B cell lymphoma. Br J Haematol.

[2] Trneny M, Verhoef G, Dyer MJ, Ben Yehuda D, Patti C, Canales M (2018). A phase II multicenter study of the anti-CD19 antibody drug conjugate coltuximab ravtansine (SAR3419) in patients with relapsed or refractory diffuse large B-cell lymphoma previously treated with rituximab-based immunotherapy. Haematologica.

[3] Chow VA, Shadman M, Gopal AK (2018). Translating anti-CD19 CAR T-cell therapy into clinical practice for relapsed/refractory diffuse large B-cell lymphoma. Blood.

[4] Neelapu SS, Locke FL, Bartlett NL, Lekakis LJ, Miklos DB, Jacobson CA (2017). Axicabtagene ciloleucel CAR T-cell therapy in refractory large B-cell lymphoma. N Engl J Med.

[5] Stirrups R (2018). CAR T-cell therapy in refractory large B-cell lymphoma. Lancet Oncol.

[6] Pan J, Niu Q, Deng B, Liu S, Wu T, Gao Z, et al. CD22 CAR T-cell therapy in refractory or relapsed B acute lymphoblastic leukemia. Leukemia. 2019. 10.1038/s41375-019-0488-7. 10.1038/s41375-019-0488-7 31110217

[7] Kusumoto S, Arcaini L, Hong X, Jin J, Kim WS, Kwong YL (2019). Risk of HBV reactivation in patients with B-cell lymphomas receiving obinutuzumab or rituximab immunochemotherapy. Blood.

[8] Kusumoto S, Tanaka Y, Mizokami M, Ueda R (2009). Reactivation of hepatitis B virus following systemic chemotherapy for malignant lymphoma. Int J Hematol.

[9] Kusumoto S, Tanaka Y, Suzuki R, Watanabe T, Nakata M, Takasaki H (2015). Monitoring of hepatitis B virus (HBV) DNA and risk of HBV reactivation in B-cell lymphoma: a prospective observational study. Clin Infect Dis.

[10] Tsutsumi Y, Yamamoto Y, Ito S, Ohigashi H, Shiratori S, Naruse H (2015). Hepatitis B virus reactivation with a rituximab-containing regimen. World J Hepatol.

[11] Sagnelli C, Pisaturo M, Calo F, Martini S, Sagnelli E, Coppola N (2019). Reactivation of hepatitis B virus infection in patients with hemo-lymphoproliferative diseases, and its prevention. World J Gastroenterol.

[12] Sarmati L, Andreoni M, Antonelli G, Arcese W, Bruno R, Coppola N (2017). Recommendations for screening, monitoring, prevention, prophylaxis and therapy of hepatitis B virus reactivation in patients with haematologic malignancies and patients who underwent haematologic stem cell transplantation-a position paper. Clin Microbiol Infect.

[13] Chavez JC, Bachmeier C, Kharfan-Dabaja MA (2019). CAR T-cell therapy for B-cell lymphomas: clinical trial results of available products. Ther Adv Hematol.

[14] Jacobson CA (2019). CD19 chimeric antigen receptor therapy for refractory aggressive B-cell lymphoma. J Clin Oncol.

[15] Dong HJ, Ni LN, Sheng GF, Song HL, Xu JZ, Ling Y (2013). Risk of hepatitis B virus (HBV) reactivation in non-Hodgkin lymphoma patients receiving rituximab-chemotherapy: a meta-analysis. J Clin Virol.

[16] Kim SJ, Hsu C, Song YQ, Tay K, Hong XN, Cao J (2013). Hepatitis B virus reactivation in B-cell lymphoma patients treated with rituximab: analysis from the Asia lymphoma study group. Eur J Cancer.

[17] Strati Paolo, Nastoupil Loretta J., Fayad Luis E., Samaniego Felipe, Adkins Sherry, Neelapu Sattva S. (2019). Safety of CAR T-cell therapy in patients with B-cell lymphoma and chronic hepatitis B or C virus infection. Blood.

[18] European Association for the Study of the Liver. Electronic address eee, European Association for the Study of the L (2017). EASL 2017 clinical practice guidelines on the management of hepatitis B virus infection. J Hepatol.

[19] Reddy KR, Beavers KL, Hammond SP, Lim JK, Falck-Ytter YT (2015). American Gastroenterological Association I. American Gastroenterological Association Institute guideline on the prevention and treatment of hepatitis B virus reactivation during immunosuppressive drug therapy. Gastroenterology.

[20] Huang H, Li X, Zhu J, Ye S, Zhang H, Wang W (2014). Entecavir vs lamivudine for prevention of hepatitis B virus reactivation among patients with untreated diffuse large B-cell lymphoma receiving R-CHOP chemotherapy: a randomized clinical trial. JAMA.

